# The role of offensive and creative priming videos in enhancing youth football players’ performance during small-sided games

**DOI:** 10.3389/fpsyg.2025.1553561

**Published:** 2025-04-03

**Authors:** Diogo Coutinho, Bruno Gonçalves, Sara Santos, Sigrid Olthof, Rafael Ballester Lengua, André Roca, Bruno Travassos

**Affiliations:** ^1^Department of Physical Education and Sports Sciences, University of Maia (UMAIA), Maia, Portugal; ^2^Research Center in Sports Sciences, Health Sciences and Human Development, CIDESD, Vila Real, Portugal; ^3^Departamento de Desporto e Saúde, Escola de Saúde e Desenvolvimento Humano, Universidade de Évora, Évora, Portugal; ^4^Comprehensive Health Research Centre (CHRC), Universidade de Évora, Évora, Portugal; ^5^Department of Sports Sciences, Exercise and Health, University of Trás-os-Montes and Alto Douro, Vila Real, Portugal; ^6^Research Institute for Sport and Exercise Sciences, Liverpool John Moores University, Liverpool, United Kingdom; ^7^Department of Physical Education and Sport Sciences, Catholic University of Valencia “San Vicente Martir”, Valencia, Spain; ^8^Research Centre for Applied Performance Sciences, Faculty of Sport, Technology and Health Sciences, St Mary’s University, London, United Kingdom; ^9^Portugal Football School, Portuguese Football Federation, Oeiras, Portugal; ^10^Department of Sports Sciences, University of Beira Interior, Covilhã, Portugal

**Keywords:** soccer, training, decision-making, physical performance, creativity

## Abstract

**Introduction:**

This study examined the effects of video-based priming interventions on youth football players’ performance prior to playing small-sided games (SSG).

**Methods:**

Twenty-four U14 players (age: 13.8 ± 0.4 years, football experience of 7.5 ± 2.3 years) participated in three conditions: (i) CONTROL (no priming), (ii) OFFENSIVE priming (a 4-minute video on progressive possession style leading to goals), and (iii) CREATIVE priming (a 4-minute video emphasizing innovative passes, dribbles, and shots). Tactical and physical performance were assessed using GPS devices, individual tactical performance using the Game Performance Evaluation Tool (GPET), and performance creativity using the CREATIVE Behavior Assessment in Team Sports (CBATS). Data were compared using the non-parametric Friedman ANOVA test.

**Results:**

The OFFENSIVE condition demonstrated reduced variability in distances to teammates (*X*^2^ = 7.00, *p* = 0.030), and increased overall external load compared to the CONTROL condition. Superior decision-making (*X*^2^ = 18.6, p < 0.001) and execution (*X*^2^ = 13.2, *p* = 0.001) in passing actions compared to both the control and creative conditions were observed. The CREATIVE condition promoted increased spatial exploration (*X*^2^ = 6.10, *p* = 0.047), and more frequent shooting attempts (*X*^2^ = 7.05, *p* = 0.029) compared to the CONTROL and OFFENSIVE conditions, and greater variability in distances to opponents compared to the CONTROL condition (*X*^2^ = 9.75, *p* = 0.008).

**Discussion:**

These findings demonstrate that video-based priming can influence tactical, technical, and creative behaviors in SSG. Coaches can leverage offensive priming to improve structured passing and positioning, while creative priming may inspire exploratory movements and innovative shooting attempts.

## Introduction

Small-sided games (SSGs) are widely recognized as training strategies in football to foster player development during training sessions, employed by coaches at various levels, across youth and professional levels (Clemente et al., 2021; Fernández-Espínola et al., 2020; Serra-Olivares et al., 2015). SSGs involve game-based formats where key elements such as the number of players, pitch size or shape, and playing rules are systematically manipulated to target specific movement behaviors and enhance performance (Davids et al., 2013). These adjustments create environments in which players’ actions and decision-making are guided by their ability to perceive and respond to uncertainty caused by their opponents’ and teammates’ positioning, ball location, and spatial references (Travassos et al., 2023). SSGs can therefore be a useful learning strategy for players to develop their physical, technical, and tactical actions influenced by task constraints and coaches’ instructions.

A substantial body of literature has examined how players adapt their positioning, technical skills, and physical performance in response to manipulated SSG conditions. For example, research has explored the effects of numerical imbalances (Gonçalves et al., 2016), pitch size (Olthof et al., 2018), or playing rules (e.g., limiting touches allowed, or restricting spaces) (Coutinho et al., 2021; Gonçalves et al., 2017; Santos et al., 2020). In addition to those task constraints, coaches themselves have an impact on the players’ performance in SSGs (Rampinini et al., 2007). More specifically, the type of instructions of the coaches has an impact on the type of actions or movements from the players. To illustrate, Baptista et al. (2018) highlighted the impact of different instructional styles on players’ movement behaviors during 7-a-side SSGs. The defensive strategy led to increased defensive behavior: reduced space exploration, an increase in technical defensive actions, and greater distances covered at low intensities compared to the control condition. Conversely, the offensive strategy resulted in greater offensive behavior: greater pitch width usage, improved passing technical performance, and increased jogging distance.

Building on this, video-based instructional styles have been widely used by coaches to provide performance feedback both in training and matches (O’Donoghue, 2006). Furthermore, video analysis is commonly used to examine opposition movement patterns. Coaches present these patterns to players (e.g., highlighting key aspects such as spaces to exploit when in possession) to guide their decision-making and on-field behavior. Thus, growing evidence suggests it can also serve as an instructional tool to prime players before engaging in tasks (Collins et al., 2023; O’Donoghue, 2006). For example, a systematic review by Zhao et al. (2022) provided insights into the effects of video-based training on anticipation and decision-making skills in football players, underscoring that video training improved response accuracy and decision-making judgments. More recently, Martinez et al. (2024) found that video use can enhance players’ ball control skills. Additionally, research indicates that players perceive video-based training as beneficial for their performance. In this regard, van Maarseveen et al. (2018) examined the impact of self-controlled video feedback on tactical skills during 3v2 SSGs, revealing that players who selected video feedback after successful trials demonstrated increased engagement and a deeper understanding of their performance. While these findings highlight the effectiveness of video in supporting learning and performance, they primarily focus on its role in explicit feedback and skill acquisition. However, less is known about how videos designed to inspire and prime specific actions influence players’ behavior in SSGs. Unlike traditional video training, which aims to refine skills through direct instruction, priming videos may implicitly shape tactical and technical behaviors. Further research is warranted to explore the immediate (acute) effects of video-based priming on players’ decision-making in football.

Using videos to guide players’ actions in subsequent performances is a priming strategy (Collins et al., 2023), as they “prime” to empower players’ performance. Priming refers to the subtle and often unconscious neural and mental activation of goals, attitudes, or concepts through external stimuli, which subsequently influence behavior (Bargh et al., 2001). This psychological mechanism has been extensively studied across various domains, including cognitive tasks, social behaviors, and sport (Shanks et al., 2013). In sport, research has shown that priming specific achievement goals or traits can enhance cooperation, accuracy, and automaticity in motor tasks (Adams et al., 2014; Greenlees et al., 2014). Priming interventions have also been found to improve the speed and accuracy of soccer dribbling tasks, emphasizing their potential to promote automatic execution of skills (Adams et al., 2014) or even enhance match-day performance. Furley and Memmert (2018) demonstrated a significant positive impact on players’ creative decision-making, as primed players generated more diverse and novel tactical solutions compared to a control group. Creativity is widely recognized as a pivotal trait in team sports, valued for its integral role in enhancing performance (Furley and Memmert, 2018). While previous findings highlight the potential of video-based priming to influence individual creativity, it remains unclear how video priming may affect player performance in SSGs, where dynamic interactions and contextual constraints play a crucial role.

This gap in the literature underscores the need for further research to examine the effectiveness of video priming within ecological and game-based frameworks, exploring its impact on individual and collective tactical and physical performance in SSGs. Thus, this study investigated the impact of video-based priming on youth football players’ performance in SSGs by comparing a standard SSG (CONTROL) with two experimental conditions: OFFENSIVE priming, which encouraged attacking play, and CREATIVE priming, that showcased skilled players executing innovative moves.

Based on the findings of Baptista et al. (2018), we hypothesize that exposure to the offensive video, which emphasizes a progressive possession-based attacking style, will lead to an increase in the distance covered at moderate intensity, reflecting movements aimed at creating space. Based on the findings from Baptista et al. (2018) and (Furley and Memmert, 2018), we expect that showing offensive videos to players will lead to greater attacking actions, whilst showing creative videos will lead to an increase in creative actions.

## Methods

### Participants

A total of 24 youth male football players participated in this study, representing an U14 regional selection team (age: 13.8 ± 0.4 years, height: 166.1 ± 11.1 cm, weight: 54.3 ± 7.6 kg, and football experience: 7.5 ± 2.3 years). The team comprised the most skilled players from seven local clubs, selected by experienced coaches, to compete in a national tournament. Players followed a regular training program consisting of three 90-min sessions per week with their respective clubs, in addition to participating in 11-a-side matches during weekends.

Only outfield players were considered in the analysis. Before the start of the study, one player was excluded due to injury or illness, and two players were excluded because they were unable to attend all data collection sessions. Written informed consent was obtained from all participants, their legal guardians, the regional football association, as well as the clubs and coaches involved. All participants were informed of their right to withdraw from the study at any time without facing any negative consequences. The study adhered to the ethical guidelines of the local university’s ethics committee and followed the principles outlined in the Declaration of Helsinki.

## Study design

The players were grouped in four balanced teams, each consisting of one goalkeeper and six outfield players. The teams were assembled by the head coach during each testing session based on the players’ individual capacities. To investigate the impact of priming, the players participated in three distinct experimental conditions; (i) CONTROL condition, which consists of practicing 6-a-side plus Goalkeeper SSG, without any priming strategy; (ii) OFFENSIVE priming, which involved a 4-min video showcasing collective plays based on possession-based style, selected for its potential to enhance positioning and technical actions like passing (e.g., videos of teams demonstrating a progressive playing style, such as Barcelona or Manchester City, where they build up play from the back through sustained possession, lasting over 20 s, and primarily relying on passing to advance and create scoring opportunities); and (iii) CREATIVE priming, featuring a 4-min video highlighting individual creative actions, including ~80-s clips of passing (e.g., Özil heel pass), dribbling (e.g., Ronaldinho flip flap movement), and shooting (e.g., Zlatan Ibrahimović acrobatic finishes).

This design was informed by research showing that exposure to creative role models can activate mental representations of creativity, fostering flexibility and fluency in decision-making and technical execution (Furley and Memmert, 2018). The video duration was informed by the work of Bargh et al. (2001), which demonstrated that nonconscious goal activation can significantly influence behavior without requiring conscious awareness. This finding supports the use of brief interventions, such as a 4-min video, to effectively prime individuals by subtly activating specific goals or behaviors. Additionally, research suggests that longer durations may attenuate priming effects due to decreased attention or cognitive saturation over time (Bargh et al., 2001; Higgins et al., 1985). Each team watched the priming videos on a single 16-inch laptop during the recovery periods between SSGs. The team viewed the same video collectively. However, although this setup is practical for a training session and on-pitch video priming, it does not control potential peer influence during video viewing. The videos were selected by two expert coaches, each with over 15 years of coaching experience, holding UEFA Pro and UEFA A Football licenses, respectively.

## Procedures

Data collection spanned over four testing sessions during the competitive period of the 2022–2023 season (January–February). The pilot session was dedicated to familiarizing players with the experimental conditions, which included CONTROL, OFFENSIVE, and CREATIVE scenarios. In this session, the players watched the same videos used in the main experiment, but with a reduced duration of 2-min, ensuring they were exposed to the experimental conditions while maintaining the same design principles. In the subsequent three sessions, players were randomly assigned to experience the conditions in varying orders (e.g., during the first session: (i) OFFENSIVE, (ii) CONTROL, (iii) CREATIVE, with the order shuffled for each subsequent session using Random.org. In the second session, the order was: (i) CONTROL, (ii) CREATIVE, (iii) OFFENSIVE, while in the third session, it was: (i) OFFENSIVE, (ii) CREATIVE, (iii) CONTROL). To maintain a repeated-measures design, teams remained consistent across all sessions; however, player composition within each team varied across testing days. This approach ensured diverse team configurations while maintaining the same structure across conditions, enhancing the generalizability of findings (Coutinho et al., 2024).

Sessions commenced with a standardized 15-min warm-up, comprising running drills, mobility exercises, and a possession-focused game (6-a-side without goals). After the warm-up, players participated in the experimental conditions. SSGs were conducted on a 64 × 43 m artificial turf pitch (length-to-width ratio ~ 1.49; relative pitch area (RPA) of 196.6 m^2^), using a 6-a-side plus goalkeeper format. This format was chosen because it is the one players have been most exposed to throughout their careers and formats larger than 4-a-side have been proposed for studying tactical behavior as it allows representation of all playing sectors (i.e., defensive, midfield, and offensive) (Aguiar et al., 2015). Furthermore, it has been widely used in research to examine tactical behavior in SSGs with youth players (Santos et al., 2020). The RPA in the study aligns with suggestions of Riboli et al. (2022) to meet physical match demands. Official 7-a-side goals (6 × 2 m) were used. Each experimental condition consisted of a single 5-min SSG, interspersed by 5-min passive recovery periods before progressing to the subsequent condition. During recovery, players viewed the respective priming videos on laptops near the sidelines before returning to play. To minimize disruptions, several balls were strategically placed around the pitch perimeter. No coaching feedback or encouragement was permitted to avoid influencing player behavior. SSGs adhered to FIFA rules, except games were restarted by the goalkeeper after a goal or when the ball exited via the end line, ensuring rapid resumption.

## Data collection

### Tactical and physical performance

Positional and physical performance metrics were captured using GPS devices (10 Hz, FieldWiz, Paudex, Switzerland), which have been validated for their reliability in tracking movements and displacements in team sports (Willmott et al., 2019). While the accuracy of FieldWiz GPS devices has not been explicitly validated from a tactical analysis perspective, previous studies have utilized these units to examine team tactical behaviors in SSGs (Coutinho et al., 2024) and simulated matches (Clemente et al., 2017), suggesting their applicability in similar contexts. Latitude and longitude data recorded by the GPS units were resampled to address potential gaps and ensure synchronization across players, then transformed into meters using the Universal Transverse Mercator (UTM) coordinate system. Dynamic positional data were analyzed for distances between each player and their nearest teammate and opponent in terms of absolute values (in meters), variability (expressed as the coefficient of variation, CV), and regularity (expressed in arbitrary units, a.u.) using the approximate entropy (ApEn) method (Pincus, 1991). ApEn is commonly applied to assess variability structures in time-series data, with vector length set at 2 and tolerance at 0.2*std (Yentes et al., 2013). The resulting ApEn values range from 0 to 2 a.u., where values near 0 indicate high regularity (e.g., players maintaining consistent distances over time), and values closer to 1 reflect greater irregularity (Gonçalves et al., 2016). Additionally, the spatial exploration index (SEI) was computed to evaluate the extent of space utilized by players during the tasks (Gonçalves et al., 2017).

Physical performance included total distance covered and distances at different speed ranges categorized as walking (0.0–3.5 km/h), jogging (3.6–14.3 km/h), running (14.4–19.8 km/h), and sprinting (>19.9 km/h) based on thresholds from prior SSG research (Coutinho et al., 2024; Coutinho et al., 2021; Pettersen and Brenn, 2019). Average speed (m/s) was also calculated.

### Game performance evaluation tool (GPET)

SSG conditions were recorded using a Panasonic NV-GS230 digital video camera positioned centrally at a 2-m height for comprehensive field coverage. Player actions were analyzed and tagged using The Play software (Metrica Sports, version 2.20.2) through a systematic notational analysis process (Coutinho et al., 2024; Coutinho et al., 2023).

Individual tactical performance was analyzed using the Game Performance Evaluation Tool (GPET), a validated framework for assessing decision-making and execution in individual tactical actions (García-López et al., 2013). For decision-making, GPET considers the adequateness of the players’ selected responses based on local information, such as teammates’ and opponents’ positioning. The analysis of execution evaluates the success or failure of an action. While the original instrument was focused on the decision-making and execution, additional technical criteria have been included to add further details to understand players’ actions (Coutinho et al., 2024; Coutinho et al., 2023). Accordingly, for ball control, the analysis included (i) execution; (ii) maintaining motor space (ability to keep the ball close enough to remain in possession), (iii) body orientation (receiving the ball while oriented to optimize the next action in relation to opponent’s goal), and (iv) adequacy of ball reception (using the correct body part for effective CONTROL). Passing performance was assessed based on (i) decision-making, (ii) execution, (iii) passing to an offensive teammate, representing the capacity to select offensive options that facilitate progression; and (iv) free teammate, which consists in the ability to pass to unmarked teammates to create space. Dribbling was also analyzed in relation to (i) decision-making, (ii) execution, (iii) deception, which assesses whether the player used body feints or sudden movements to mislead opponents, and (iv) change of rhythm, evaluating the player’s ability to change speed to overcome the opposition. GPET variables were expressed as the percentage of successful decisions relative to the total number of actions performed (e.g., the motor execution for the dribble was coded as: successful dribbles/[total dribbles]).

A total of 1,642 actions (ball control: *n* = 383; passing: *n* = 932; dribbling: *n* = 327) were analyzed by an expert analyst with over 15 years of training and match analysis experience. Intra-observer reliability (10% of the sample reanalyzed) demonstrated high agreement (Coutinho et al., 2023; Serra-Olivares et al., 2015) with Kappa values ranging from 0.72 to 0.89 (O'Donoghue, 2010).

### Creative components measured using the CBATS

The CREATIVE Behavior Assessment in Team Sports (CBATS) use the same video recording setup as GPET. This framework is designed to assess creativity within ball possession contexts, focusing on four primary components: fails, attempts, fluency, and versatility (Santos et al., 2023; Santos et al., 2017). The recorded footage was analyzed using customized notational analysis software, with creative components tagged and quantified according to predefined criteria: (i) Fails, represented unsuccessful actions performed using standardized techniques (e.g., a misdirected pass with the inside of the foot or a failed dribble to the side); (ii) Attempts, measured the frequency of unsuccessful but innovative actions, highlighting efforts to explore non-standardized techniques even if they did not succeed (e.g., an unconventional pass or dribble that failed); (iii) fluency, measured as the number of successful actions performed using standard techniques (e.g., passing with the inside of the foot); and (iv) versatility, representing the variety of non-standardized successful actions (e.g., a backheel pass or an overhead kick).

The CBATS framework allows for nuanced insights into how players generate and execute creative solutions under game constraints (Santos et al., 2023; Santos et al., 2017). To ensure data reliability, 20% of the video samples were reanalyzed 1 week later by the same performance analyst, who had significant expertise (i.e., 15-years of experience) in notational analysis and creative behavior assessment. The intraclass correlation coefficients (ICCs) for the reanalyzed data were consistently high, with Kappa values ranging from 0.83 to 0.92 (O'Donoghue, 2010).

### Statistical analysis

Descriptive analysis was conducted using means and standard deviations. The Shapiro–Wilk test was applied to evaluate outliers and assess normality assumptions for all collected data. Considering that non-normal distribution of the data, the non-parametric Friedman ANOVA was utilized. Pairwise comparisons were performed using the Durbin-Conover test. Statistical significance was set at *p* ≤ 0.05, and all calculations were carried out using the Jamovi Project software (Version 1.2, 2020). Additionally, differences in means were reported with 95% confidence intervals (raw data), and Cohen’s d was calculated to assess effect sizes. Effect size thresholds were categorized as follows: 0.0–0.19 (trivial), 0.20–0.49 (small), 0.50–1.19 (moderate), 1.20–1.99 (large), and ≥2.0 (very large) (Hopkins et al., 2009).

## Results

### Tactical and physical performance

**Figure 1:**
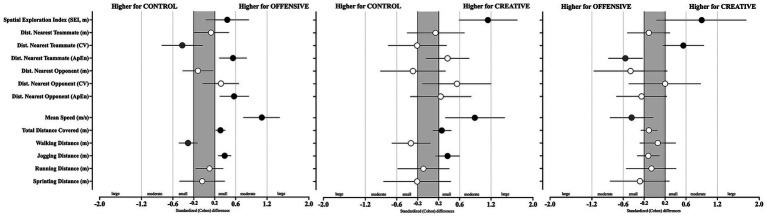
Cohen *d* for positioning and time-motion variables according to SSG condition (CONTROL, OFFENSIVE, and CREATIVE). Error bars indicate uncertainty in the true mean changes with 95% confidence intervals. Black or gray marks indicates a positive effect toward the corresponding condition, while 0 would indicate unclear effects.

Tactical and physical performance results across the various SSG conditions (i.e., CONTROL vs. OFFENSIVE; CONTROL vs. CREATIVE; OFFENSIVE vs. CREATIVE) are presented in Table 1 and Figure 1. Statistically significant effects were observed for the SEI (*X*^2^ = 6.10, *p* = 0.047), coefficient of variation (CV, *X*^2^ = 7.00, *p* = 0.030), and ApEn for distance to the nearest teammate (*X*^2^ = 10.3, *p* = 0.006), as well as distance to the nearest opponent (CV, *X*^2^ = 9.75, *p* = 0.008).

**Table 1:** Inferential and descriptive statistics from tactical and physical related variables from the different SSG conditions (CONTROL, OFFENSIVE, CREATIVE).

Variables	CONTROL	OFFENSIVE	CREATIVE	Difference in means (raw ± 95% CI)			*p*
	(M + SD)	(M + SD)	(M + SD)	CONTROL vs. OFFENSIVE	CONTROL vs. CREATIVE	OFFENSIVE vs. CREATIVE	
Tactical variables							
Spatial exploration index (SEI, m)	5.62 ± 0.97	6.17 ± 1.04	7.11 ± 1.52	0.55; ±0.51	1.43; ±0.69	1.12; ±1.07	**0.047** ^**b,c**^
Dist. nearest teammate (m)	7.08 ± 1.76	7.30 ± 1.50	6.84 ± 1.64	0.23; ±0.56	0.24; ±0.92	−0.19; ±0.68	0.130
Dist. nearest teammate (CV)	40.60 ± 13.29	36.37 ± 7.31	42.9 ± 7.42	−4.22; ±3.95	−2.08; ±5.55	5.53; ±3.94	**0.030** ^**a**^
Dist. nearest teammate (ApEn)	0.13 ± 0.06	0.16 ± 0.04	0.14 ± 0.05	0.03; ±0.01	0.02; ±0.02	−0.03; ±0.02	**0.006** ^**a,c**^
Dist. nearest opponent (m)	5.01 ± 1.39	4.86 ± 1.19	4.55 ± 1.10	−0.15; ±0.37	−0.36; ±0.79	−0.58; ±0.89	0.311
Dist. nearest opponent (CV)	43.86 ± 6.75	46.38 ± 8.66	48.71 ± 7.58	2.51; ±2.71	4.33; ±5.23	1.57; ±5.42	**0.008** ^**a,b**^
Dist. nearest opponent (ApEn)	0.16 ± 0.06	0.19 ± 0.05	0.17 ± 0.05	0.03; ±0.02	0.01; ±0.03	−0.01; ±0.03	0.275
Physical variables							
Mean speed (m/s)	4.55 ± 1.04	5.49 ± 0.63	5.20 ± 0.75	0.94; ±0.30	0.76; ±0.48	−0.38; ±0.35	**<0.001** ^**a,b**^
Total distance covered (m)	255.64 ± 135.18	296.49 ± 115.15	300.17 ± 133.74	40.85; ±12.35	34.86; ±23.18	−14.37; ±20.50	**<0.001** ^**a,b**^
Walking distance (m)	45.50 ± 13.87	40.67 ± 16.64	43.28 ± 15.09	−4.83; ±2.77	−5.15; ±5.76	0.94; ±5.36	0.197
Jogging distance (m)	195.93 ± 117.65	239.96 ± 95.03	240.91 ± 114.08	44.04; ±13.55	41.97; ±26.08	−13.60; ±23.76	**<0.001** ^**a,b,c**^
Running distance (m)	13.72 ± 14.80	15.11 ± 14.97	15.55 ± 16.50	1.60; ±4.09	−1.45; ±7.84	−1.03; ±7.58	0.549
Sprinting distance (m)	0.85 ± 3.12	0.75 ± 2.33	0.43 ± 1.16	−0.10; ±1.05	−0.51; ±1.54	−0.68; ±1.37	0.971

(a) Statistically significant differences between CONTROL and OFFENSIVE; (b) Statistically significant differences between CONTROL and CREATIVE; (c) Statistically significant differences between OFFENSIVE and CREATIVE. Bold values indicate statistically significant differences (*p* < 0.05) between conditions.

Regarding the SEI, higher values were found in the CREATIVE condition compared to both the CONTROL (*p* = 0.024; moderate higher, Cohen’s *d* [95% CL] = 1.14 [0.59; 1.7]) and OFFENSIVE conditions (*p* = 0.036; moderate higher, ES = 0.9 [0.04; 1.75]). For distances to the nearest teammate, lower variability was observed during the OFFENSIVE condition compared to the CONTROL (distance to the nearest teammate CV, *p* = 0.008; small lower, ES = −0.42 [−0.81; −0.03]). In contrast, higher variability in the distance to the nearest opponent was found in the OFFENSIVE condition (*p* = 0.022, unclear effects, ES = 0.32 [−0.03; 0.66]). The CONTROL condition also revealed lower variability in the distance to the nearest opponent (*p* = 0.022 unclear effects, ES = 0.55 [−0.11; 1.2]) compared to the CREATIVE condition.

In terms of regularity (i.e., ApEn) for the distance to nearest teammate, lower regularity was observed during the OFFENSIVE condition compared to both the CONTROL (*p* = 0.001; small lower, ES = 0.55 [0.29; 0.81]) and CREATIVE conditions (*p* = 0.030; moderate lower, ES = −0.56 [−0.88; −0.23]).

From the physical performance perspective, statistically significant effects were identified for mean speed (*X*^2^ = 18.1, *p* = <0.001), total distance covered (*X*^2^ = 18.1, *p* = <0.001) and jogging distance (*X*^2^ = 15.3, *p* = <0.001). The CONTROL condition showed lower values for mean speed (vs OFFENSIVE, *p* < 0.001; moderate lower, ES = 1.1 [0.75; 1.44]; vs. CREATIVE, *p* = 0.001; moderate lower, ES = 0.89 [0.33; 1.46]), total distance covered (vs OFFENSIVE, *p* < 0.001; small lower, ES = 0.31 [0.22; 0.4]; vs. CREATIVE, *p* = 0.001; small lower, ES = 0.26 [0.09; 0.44]), and jogging distance (vs OFFENSIVE, *p* < 0.001; small lower, ES = 0.39 [0.27;0.51]; vs. CREATIVE, *p* = 0.014; small lower, ES = 0.37 [0.14;0.6]) compared to both the OFFENSIVE and CREATIVE conditions. Additionally, the CREATIVE condition revealed a higher jogging distance than the OFFENSIVE condition (*p* = 0.046; unclear effects98, ES = −0.12 [−0.33;0.09]).

### Game performance evaluation tool (GPET)

**Figure 2:**
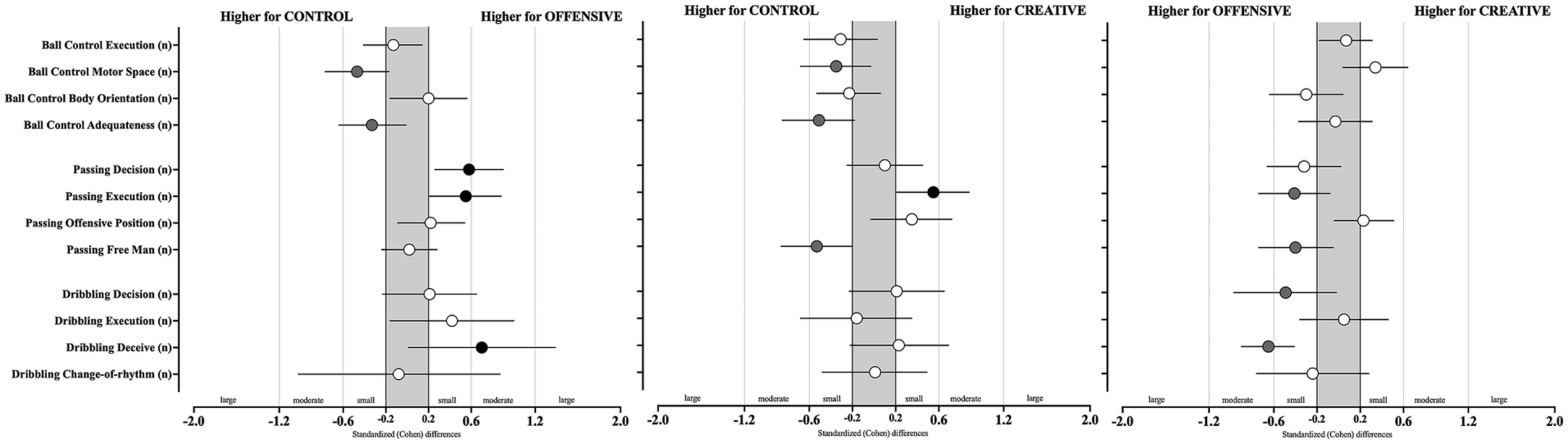
Cohen *d* for GPET variables according to SSG condition (CONTROL, OFFENSIVE, and CREATIVE). Error bars indicate uncertainty in the true mean changes with 95% confidence intervals. Black or gray marks indicates a positive effect toward the corresponding condition, while 0 would indicate unclear effects.

Results from GPET in the SSG conditions (i.e., CONTROL vs. OFFENSIVE; CONTROL vs. CREATIVE; OFFENSIVE vs. CREATIVE) are presented in Table 2 and Figure 2. Statistically significant effects were identified for ball control in the motor space (*X*^2^ = 8.14, *p* = 0.004), body orientation (*X*^2^ = 6.04, *p* = 0.049), passing decision-making (*X*^2^ = 18.6, *p* = <0.001), passing execution (*X*^2^ = 13.2, *p* = 0.001), passing to teammate in most offensive position (*X*^2^ = 14.0, *p* = <0.001), and dribbling deceive (*X*^2^ = 9.79, *p* = 0.007). Accordingly, higher ability to control the ball in the motor space was found during the CONTROL condition when compared to the OFFENSIVE condition (*p* = 0.004; unclear effects, ES = −0.47 [−0.77; −0.17]). Higher body orientation toward the opponent’s goal was observed in the OFFENSIVE condition compared to the CREATIVE condition (*p* = 0.004; unclear effects, ES = −0.3 [−0.64; 0.04]). From the passing perspective, better passing decision (vs CONTROL, *p* < 0.001; moderate higher, ES = 0.58 [0.26; 0.9]; vs. CREATIVE, *p* < 0.001; unclear effects, ES = −0.32 [−0.66; 0.02]) and execution were found during the OFFENSIVE condition when compared to both the CONTROL and CREATIVE conditions. In terms of the ability to pass to players in most OFFENSIVE positions, lower values were identified during the CONTROL when compared to both the OFFENSIVE (*p* 0.011; unclear effects, 0.22 [−0.09; 0.54]) and the CREATIVE conditions (*p* < 0.001; unclear effects, 0.23 [−0.04; 0.51]). Finally, from the dribbling deceive perspective, higher mean values were found during the OFFENSIVE condition compared to both the CONTROL (*p* = 0.032; moderate higher, ES = 0.7 [0.01; 1.39]) and CREATIVE (*p* = 0.001; moderate higher, ES = −0.65 [−0.9; −0.41]) conditions.

**Table 2:** Inferential and descriptive statistics from GPET related variables from the different SSG conditions (CONTROL, OFFENSIVE, CREATIVE).

Variables	CONTROL	OFFENSIVE	CREATIVE	Difference in means (raw ± 95% CI)			*p*
	(M + SD)	(M + SD)	(M + SD)	CONTROL vs. OFFENSIVE	CONTROL vs. CREATIVE	OFFENSIVE vs. CREATIVE	
Ball CONTROL-related variables							
Execution (n)	0.93 ± 0.22	0.90 ± 0.20	0.87 ± 0.23	−0.03; ±0.06	−0.07; ±0.07	0.01; ±0.05	0.148
Motor space (n)	0.94 ± 0.13	0.85 ± 0.23	0.87 ± 0.23	−0.10; ±0.06	−0.07; ±0.07	0.07; ±0.06	**0.017** ^**a**^
Body orientation (n)	0.51 ± 0.35	0.56 ± 0.33	0.46 ± 0.37	0.07; ±0.13	−0.08; ±0.1	−0.11; ±0.12	**0.049** ^**c**^
Adequateness (n)	0.95 ± 0.13	0.89 ± 0.22	0.84 ± 0.24	−0.07; ±0.06	−0.10; ±0.07	−0.01; ±0.07	0.175
Pass-related variables							
Passing decision (n)	0.83 ± 0.15	0.9 ± 0.17	0.85 ± 0.16	0.09; ±0.05	0.02; ±0.06	−0.05; ±0.05	**0.049** ^**a,c**^
Passing execution (n)	0.67 ± 0.17	0.79 ± 0.30	0.69 ± 0.23	0.13; ±0.08	0.02; ±0.07	−0.10; ±0.08	**0.001** ^**a,c**^
Passing offensive position (n)	0.44 ± 0.26	0.47 ± 0.24	0.54 ± 0.31	0.06; ±0.09	0.10; ±0.10	0.06; ±0.08	**<0.001** ^**a,b**^
Passing free man (n)	0.74 ± 0.20	0.74 ± 0.27	0.64 ± 0.29	0.00; ±0.07	−0.14; ±0.09	−0.10; ±0.09	0.071
Dribbling-related variables							
Dribbling decision (n)	0.71 ± 0.32	0.70 ± 0.39	0.73 ± 0.39	0.03; ±0.23	0.08; ±0.17	−0.19; ±0.18	0.091
Dribbling execution (n)	0.43 ± 0.38	0.58 ± 0.46	0.42 ± 0.42	0.18; ±0.25	−0.07; ±0.22	0.02; ±0.18	1.000
Dribbling deceive (n)	0.27 ± 0.38	0.65 ± 0.38	0.37 ± 0.39	0.28; ±0.27	0.09; ±0.18	−0.26; ±0.10	**0.049** ^**a,c**^
Dribbling change-of-rhythm (n)	0.26 ± 0.37	0.17 ± 0.31	0.22 ± 0.37	−0.03; ±0.34	0.00; ±0.18	−0.09; ±0.19	0.199

(a) Statistically significant differences between CONTROL and OFFENSIVE; (b) Statistically significant differences between CONTROL and CREATIVE; (c) Statistically significant differences between OFFENSIVE and CREATIVE. Bold values indicate statistically significant differences (*p* < 0.05) between conditions.

### CREATIVE behavior assessment in team sports (CBATS)

**Figure 3:**
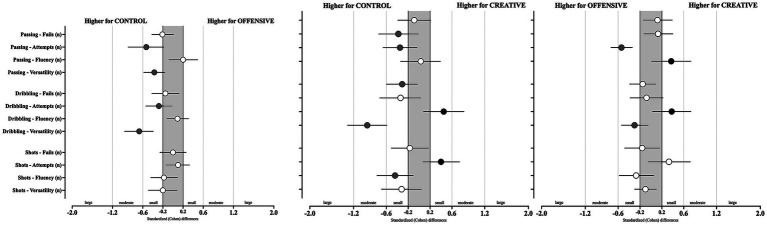
Cohen *d* for creative components variables according to SSG condition (CONTROL, OFFENSIVE, and CREATIVE). Error bars indicate uncertainty in the true mean changes with 95% confidence intervals. Black or gray marks indicates a positive effect toward the corresponding condition, while 0 would indicate unclear effects.

The creative component performances’ differences between SSG conditions (i.e., CONTROL vs. OFFENSIVE; CONTROL vs. CREATIVE; OFFENSIVE vs. CREATIVE) are presented in Table 3 and Figure 3. Statistically significant effects were identified for passing: attempts (*X*^2^ = 11.1, *p* = 0.004), fluency (*X*^2^ = 9.75, *p* = 0.008), and versatility (*X*^2^ = 11.8, *p* = 0.003), while in the dribbling attempts (*X*^2^ = 5.83, *p* = 0.054) and versatility (*X*^2^ = 35.0, *p* = <0.001), the shooting attempts (*X*^2^ = 7.05, *p* = 0.029) and fluency (*X*^2^ = 8.34, *p* = 0.015). A higher number of attempts were identified in the CONTROL condition than in the OFFENSIVE (*p* = 0.001; moderate higher, ES = −0.53 [−0.89; −0.18]) and CREATIVE (*p* = 0.0014; small higher, ES = −0.38 [−0.74; −0.01]). As regard to passing fluency, higher mean values were identified in the CONTROL when compared to the CREATIVE (*p* = 0.028; small effects, ES = −0.35 [−0.66; −0.04]), as well as in the OFFENSIVE when compared to the CREATIVE (*p* = 0.002; moderate effects, ES = −0.54 [−0.73; −0.34]). The CONTROL presented statistically significant higher passing versatile values than the OFFENSIVE (*p* < 0.001; moderate higher, ES = −0.37 [−0.58; −0.16]). A higher number of dribbling attempts was found in the CONTROL condition compared to the CREATIVE condition (*p* = 0.021; unclear effects, ES = −0.34 [−0.72; 0.03]). Additionally, the CONTROL condition had higher dribbling versatility values than both the OFFENSIVE (*p* < 0.001; moderate higher, ES = −0.67 [−0.96; −0.39]) and CREATIVE (*p* < 0.001; moderate higher, −0.95 [−1.31; −0.59]) conditions, while in addition, the OFFENSIVE also presented higher values than the CREATIVE (*p* < 0.054; small higher, ES = −0.3 [−0.54; −0.05]).

**Table 3:** Inferential and descriptive statistics from creative-components related variables from the different SSG conditions (CONTROL, OFFENSIVE, CREATIVE).

Variables	CONTROL	OFFENSIVE	CREATIVE	Difference in means (raw ± 95% CI)			*p*
	(M + SD)	(M + SD)	(M + SD)	CONTROL vs. OFFENSIVE	CONTROL vs. CREATIVE	OFFENSIVE vs. CREATIVE	
Pass-related variables							
Fails (n)	0.58 ± 1.08	0.38 ± 1.04	0.50 ± 0.82	−0.21; ±0.22	−0.09; ±0.30	0.13; ±0.27	0.139
Attempts (n)	0.21 ± 0.41	0.04 ± 0.20	0.08 ± 0.28	−0.17; ±0.11	−0.12; ±0.11	0.04; ±0.08	**0.004** ^**a,b**^
Fluency (n)	2.54 ± 2.83	3.00 ± 2.08	1.79 ± 1.56	0.46; ±0.64	−0.78; ±0.70	−1.21; ±0.44	**0.001** ^**b,c**^
Versatility (n)	0.25 ± 0.44	0.04 ± 0.20	0.25 ± 0.83	−0.21; ±0.12	0.01; ±0.21	0.21; ±0.20	**0.001** ^**a**^
Dribbling-related variables							
Fails (n)	0.33 ± 0.63	0.25 ± 0.55	0.17 ± 0.47	−0.08; ±0.15	−0.18; ±0.16	−0.08; ±0.14	0.074
Attempts (n)	0.21 ± 0.41	0.11 ± 0.32	0.08 ± 0.28	−0.10; ±0.09	−0.12; ±0.13	−0.03; ±0.10	**0.05** ^**b**^
Fluency (n)	0.38 ± 0.57	0.46 ± 0.82	0.79 ± 1.13	0.08; ±0.19	0.40; ±0.32	0.33; ±0.31	0.303
Versatility (n)	0.58 ± 0.76	0.21 ± 0.53	0.04 ± 0.20	−0.38; ±0.16	−0.53; ±0.2	−0.17; ±0.14	**<0.001** ^**a,b,c**^
Shooting-related variables							
Fails (n)	0.08 ± 0.28	0.08 ± 0.28	0.04 ± 0.20	0.00; ±0.07	−0.04; ±0.09	−0.04; ±0.08	0.472
Attempts (n)	0.04 ± 0.20	0.07 ± 0.26	0.17 ± 0.38	0.03; ±0.07	0.12; ±0.10	0.10; ±0.11	**0.029** ^**b,c**^
Fluency (n)	0.42 ± 0.50	0.33 ± 0.47	0.21 ± 0.41	−0.08; ±0.12	−0.21; ±0.15	−0.13; ±0.15	**0.015** ^**b**^
Versatility (n)	0.04 ± 0.20	0.01 ± 0.12	0.00 ± 0.00	−0.03; ±0.04	−0.04; ±0.05	−0.01; ±0.03	0.097

(a) Statistically significant differences between CONTROL and OFFENSIVE; (b) Statistically significant differences between CONTROL and CREATIVE; (c) Statistically significant differences between OFFENSIVE and CREATIVE. Bold values indicate statistically significant differences (*p* < 0.05) between conditions.

Finally, a greater number of shooting attempts was identified in the CREATIVE condition compared to both the CONTROL (*p* = 0.011; small higher, ES = 0.4 [0.07; 0.74]) and OFFENSIVE conditions (*p* = 0.047; unclear effects, ES = 0.33 [−0.05; 0.72]). Despite that, a greater number of fluency actions were found during the CONTROL condition when compared to the CREATIVE condition (*p* = 0.004; moderate higher, ES = −0.44 [−0.77; −0.11]).

## Discussion

This study explored the effects of video-based priming on youth football players’ performance during SSGs. Specifically, it compared a standard SSG (CONTROL condition) with two experimental conditions: one involving exposure to a video with offensive actions (OFFENSIVE priming) and another showcasing talented players. As expected, the use of video priming strategies influenced players’ physical performance and technical-tactical behavior during SSGs. Accordingly, the OFFENSIVE priming condition resulted in less variability but more irregular distances to the nearest teammate, alongside greater external load compared to the CONTROL condition. Notably, the OFFENSIVE condition had the most pronounced impact on GPET variables, demonstrating improved passing decision-making and execution, enhanced body orientation during ball control (i.e., ability to receive the ball facing opposition target), and greater ability to pass to teammates in the most offensive positions (i.e., in positions closer to opponents’ target). Additionally, the CREATIVE priming condition promoted greater spatial exploration, which led to increased external load compared to the CONTROL condition. However, despite expectations of enhanced technical creativity, this condition primarily influenced shooting attempts, which were more frequent than in other conditions. Creative passing and dribbling performance, however, did not show significant improvement.

### Effects of OFFENSIVE priming on players’ performance during SSGs

Over the past decades, the strategic approach to ball possession in football has evolved significantly. Earlier tactics emphasized shorter ball possessions and quick transitions (Tenga and Sigmundstad, 2011), while modern football increasingly values progressive possession strategies involving multiple players and prioritizing ball circulation. These strategies enhance the likelihood of creating goal-scoring opportunities through collaborative play and a greater frequency of passes (Almeida, 2019; Kempe et al., 2014; Taha and Ali, 2023). During the OFFENSIVE condition, players were exposed to offensive actions demonstrated by high-level teams, characterized by progressive ball possession involving multiple coordinated actions leading to goal-scoring opportunities. This condition aimed to prime players to advance collectively on the pitch and develop effective offensive strategies. The OFFENSIVE condition appeared to influence players’ perception of spatial relationships, as evidenced by a reduction in variability in the distance to the nearest teammate. This adaptive behavior may reflect a shared understanding among players that maintaining stable inter-player distances facilitates collective progression and goal creation. Spatial relations between players and teams affect passing success (Travassos et al., 2023), and thus, decreasing the variability in players’ distances may have led to more functional behavior. While not directly related to this study, Santos et al. (2020) reported that players also decreased variability in their distance from teammates to improve passing performance when different ball types were used.

While higher regularity is often associated with greater stability and functionality, it is important to recognize that in dynamic team sports, functional behavior does not always equate to increased regularity. Instead, it may reflect an optimal balance between adaptability and structure. In this context, reduced regularity may signify a more fluid, less predictable movement pattern, which can be advantageous in offensive play. For instance, evidence has shown inverse trends, where an increase in one variable (e.g., distance variability) corresponds to a decrease in another (e.g., distance regularity), suggesting that these spatial dynamics are interdependent (Coutinho et al., 2024; Santos et al., 2020; Santos et al., 2023). Therefore, in the OFFENSIVE condition, the observed decrease in both variability and regularity appears to reflect a flexible yet coordinated movement strategy (Seifert et al., 2013).

The passing performance, measured by GPET, revealed superior decision-making and execution in the OFFENSIVE condition compared to both the CONTROL and CREATIVE conditions. Players in the OFFENSIVE condition demonstrated a greater ability to passing to a teammate that facilitated progression toward the goal than those in the CONTROL condition. Such results are likely related to the capacity of attacking teams to better manage available space compared to teams in the other conditions, as evidenced by the stability in the distance to the nearest teammate. That is, players exhibited lower variability in their spatial relationships with both teammates during the OFFENSIVE condition.

Considering that players’ distance and angles are key informational sources that influence passing decision-making and execution in team sports (Travassos et al., 2023; Vilar et al., 2014), it is possible that lower variability promoted better awareness of players’ positioning, consequently enhancing their passing performance. In fact, the videos shown during this condition emphasized teams using positional play, a style of play that relies on occupying key spaces and moving the ball to manipulate defenders and exploit openings. In this context, lower variability may have allowed players to more easily identify teammates and maintain structured passing networks, whereas reduced regularity may have facilitated creative exploration of available space in response to defensive adjustments. This finding highlights the collective behavior of the team, where passing sequences are used not only to maintain possession but also to create goal-scoring opportunities. However, it is important to acknowledge that different playing styles can lead to distinct variability and regularity patterns, each being effective under different tactical circumstances. For example, a direct-play approach might favor more irregular, less structured movement patterns, emphasizing quick transitions and verticality to bypass defensive structures. Alternatively, the observed improvements in offensive play may not solely reflect enhanced attacking decision-making and execution but rather a shift in attentional focus. Players may have prioritized offensive actions while deprioritizing defensive responsibilities, allowing the team in possession to find better offensive solutions with greater fluidity and efficiency as defensive constraints were less emphasized.

Additionally, following the OFFENSIVE priming, players improved their body orientation toward the opponent’s goal and their dribbling deception skills (i.e., the ability to use the body to simulate movements that guide the opponent’ toward a different direction than the one intended). These improvements likely contributed to extended decision-making time, leading to better passing performances (Coutinho et al., 2024). Interestingly, despite attempting fewer passes and displaying reduced versatility, players demonstrated higher levels of creative fluency. This behavior suggests a task-oriented focus, likely influenced by the possession-based style presented in the priming videos. Previous studies indicate that priming interventions, such as exposure to task-relevant visual stimuli, can activate mental representations aligned with specific objectives, fostering behaviors that prioritize effectiveness and simplicity (Adams et al., 2014; Greenlees et al., 2014). The observed style in this study emphasized minimal ball touches and a high pass frequency per possession, with the standard pass emerging as the preferred strategy to achieve collective goals. Such adaptations align with the concept that priming can direct attention toward optimal task performance, reducing exploratory actions in favor of more structured, goal-oriented behaviors.

From a physical perspective, the OFFENSIVE condition induced a greater external load, particularly in terms of meters covered per second, compared to both the CONTROL and CREATIVE conditions. Players also covered greater total distances and exhibited increased walking and jogging activity relative to the CONTROL condition. These physical demands may reflect the movement patterns required to maintain low variability in inter-player distances while achieving positive passing outcomes. Players appeared to adapt by moving strategically to create space and open passing lanes. Consequently, players may have increased their pace while maintaining a high passing rate to increase their chances of progressing on the pitch and creating goal-scoring opportunities.

### Effects of CREATIVE priming on players’ performance during SSGs

Creative players possess the unique ability to disrupt and destabilize entire systems, such as an opponent’s defensive strategy, through their actions (Santos et al., 2023; Santos et al., 2017). These actions are characterized by their originality, unpredictability, and effectiveness, often leading to game-changing moments (Furley and Memmert, 2018). By executing innovative and unconventional movements, creative athletes not only challenge the opposition but also inspire dynamic and adaptive play, elevating the overall quality of performance. As result, a wide body of research started to explore which strategies can be used to improve the players’ creative behavior (Roca and Ford, 2021; Santos et al., 2023; Santos et al., 2017). While contextual factors, such as enriched environments (e.g., Skills4Genius is a sports training program grounded in variability, creative thinking, diversified practice, and physical literacy, designed to promote creative behavior in youth) are likely to contribute to improve players’ creativity, other strategies have recently been suggested, such as priming. A pioneering study by Furley and Memmert (2018) demonstrated the potential of priming to enhance sports creativity through exposure to creative role models. Building on previous findings, our study advances the understanding of priming by investigating its impact on players’ positioning, time-motion analysis, and technical and creative performance during SSGs.

Players in this study exhibited increased spatial exploration (i.e., SEI) during the CREATIVE condition compared to the CONTROL and OFFENSIVE conditions. The SEI quantifies the area of the pitch explored by players, with greater values indicating movement across a wider variety of zones. Interestingly, despite the increased SEI in the CREATIVE condition, total displacement (total distance covered) did not significantly differ between the CREATIVE and OFFENSIVE or CONTROL conditions. This indicates that increased SEI does not necessarily equate to greater overall movement volume. Instead, it suggests a redistribution of movement patterns, where players in the CREATIVE condition covered space more diversely without necessarily increasing their total distance traveled. This is particularly relevant in the context of creativity in football, as it implies that players in the CREATIVE condition may have been utilizing movement strategies that prioritize positional variability rather than linear displacement. In fact, this adaptive behavior may stem from players’ intentions to dynamically explore the environment and create space, allowing more time for decision-making and action exploration. These findings align with ecological theory of perception (Gibson, 1986), which posits that exploratory movements are fundamental to perceiving affordances and adapting to the game environment. Furthermore, these results are consistent with previous research (Coutinho et al., 2018), which shows a relationship between improved SEI and creativity after a 10-week practice intervention. In practical terms, increased exploratory behavior reduces predictability, making it more challenging for opponents to anticipate actions. From the creative behavior perspective, this spatial exploration seems essential for breaking defensive structures and fostering goal-scoring opportunities (Santos et al., 2016).

Greater spatial exploration in the CREATIVE condition likely explains the higher variability in the distance to the nearest opponent(s), as it disrupts the alignment between attackers and defenders. Furthermore, greater space exploration was accompanied by an increase in players’ external load compared to the CONTROL condition, as well as a higher jogging distance compared to the OFFENSIVE condition. These results align with previous studies exploring SEI under different SSG conditions, where higher SEI values were associated with increased external load (Coutinho et al., 2024; Santos et al., 2020).

Contrary to the current hypothesis, the CREATIVE condition did not encourage players to explore creative actions. Specifically, players in this condition attempted fewer passes and demonstrated reduced dribbling versatility compared to the CONTROL condition. Although the priming videos emphasized individual creative actions, such as innovative passes, dribbles, and shots, this emphasis did not translate into increased variability or adaptability in passing and dribbling behaviors. In other words, players did not exhibit a wider range of passing techniques or attempt more diverse dribbling patterns, suggesting that the priming did not enhance motor exploration in these specific technical actions. It is possible that the 4-min priming video was insufficient in duration to elicit meaningful creative behaviors, particularly in the absence of verbal guidance from a coach to enhance the priming effect. These findings are inconsistent with those of Furley and Memmert (2018), who found evidence that priming can enhance creativity. However, the complexity of the tasks in each study was markedly different. While Furley and Memmert (2018) used a computer-based decision-making task with LED-based stimuli, our study involved contextualized football practice using SSGs, where players had to perceive, decide, and execute actions under dynamic and multidimensional constraints. The complexity of this environment may have diluted the acute effects of the creative primes, as players prioritized achieving task success over exploratory or unconventional actions (Bargh et al., 2001). Additionally, it is possible that players did not focus their attention on passing and shooting actions per se, which may have limited the influence of the primes on their behavior (Furley and Memmert, 2018). This highlights the importance of ensuring that priming stimuli align with participants’ perceptions and expectations. Furthermore, as Cesario et al. (2010) suggested, priming effects may only emerge in specific contexts. In this case, players may have interpreted the task primarily as a team-oriented exercise, reducing the applicability of the individually focused creative primes. To address this limitation, future studies should explore whether priming for individual creativity (e.g., dribbling) is more effective in 1-vs-1 SSG situations, which may provide a more appropriate context for evaluating the transfer of creative priming to on-field behavior.

Although the CREATIVE priming did not enhance creative passing or dribbling, it positively influenced shooting attempts. This improvement may be linked to players increased spatial exploration and greater variability in their distance from the nearest defender. These adaptive behaviors likely afforded attackers more opportunities to exploit open spaces and attempt novel shooting patterns. By exploring a broader range of spaces and maintaining greater variability in proximity to defenders, players may have perceived more opportunities to attempt creative shots. Additionally, this effect might be explained by the structure and sequencing of the priming videos. The final section of the creative video emphasized scoring innovative goals, and it is plausible that this information, being most recent, remained fresh in the players’ minds during gameplay. Research on recency effects in priming (Bargh et al., 2001; Cesario et al., 2010) suggests that the most recently presented primes can have a stronger influence on subsequent behavior, particularly when these primes align with task-specific goals. In this case, the focus on creative goal-scoring may have directly influenced players to experiment with new shooting actions. Despite these findings, the most significant effects were observed in SEI and external load.

Recent studies have supported the use of video as pedagogical tool to develop players’ behaviors (Zhao et al., 2022). Coaches may empower learning opportunities by using videos entailing teams grounded on progressive possession to improve players’ passing performance. The results from this study shed light on the impact that information prior to the task may have on players’ performance. More specifically, the type of instructions impacts offensive and create movements and actions, which aligns with the impact of different verbal instructions in SSGs (Baptista et al., 2018). Therefore, the results from the current study and Baptista et al. (2018) suggests that coaches should carefully consider the type of information (i.e., video or verbal instruction) provided to players before a training task, as it is likely to shape players’ decision-making and actions.

### Limitations

While this study highlights the potential impact of video-based priming on youth football performance during SSGs, several limitations must be acknowledged. First, each condition (i.e., CONTROL, OFFENSIVE, and CREATIVE) was randomly performed on each day of the three testing sessions, which may have introduced carryover effects, as one condition could have influenced performance in subsequent conditions. To mitigate this, future studies should consider implementing one condition per day and potentially aligning withs specific game phases. For instance, future research could provide a more comprehensive understanding by differentiating between in-possession and out-of-possession phases while linking this analysis to teams’ regularity and variability patterns. This approach could help clarify the tactical implications of these movement patterns and their role in performance outcomes, particularly in structured tactical sequences such as building up from the back. Additionally, research suggests that priming effects can be both time-dependent (Bargh et al., 2001) and context-specific (Cesario et al., 2010). This implies that the timing of priming and the specific nature of the task may play critical roles in determining its effectiveness. Future studies should explore how variations in the video duration (i.e., 4, 6 or 8-min) and timing (i.e., immediately before the SSG or with a 3-or 6-min rest period between the video and SSG) influence player performance. Furthermore, the duration of active play should be controlled to ensure fair comparisons across conditions. Future research should account for SSG effective playing time (i.e., time the ball is in play), individual time in possession, and time spent in different tactical phases to gain deeper insights into how priming affects decision-making and movement behaviors. These additional temporal variables would provide a clearer perspective on the extent to which players are actively engaged in the game and how they allocate their efforts in response to priming stimuli.

Individual differences such as playing experience, age, and gender are also likely to influence responses to priming. Gender differences, in particular, may play a role, as cultural and experiential factors could shape how male and female players perceive and respond to priming stimuli. Finally, the influence of tactical formations on priming effectiveness remains unexplored. Since different formations impose distinct cognitive and spatial demands on players, future studies should analyze how priming interacts with structured tactical settings (e.g., positional play, high-pressing formations, or defensive blocks) to refine its application in training and competition. A broader understanding of priming’s effectiveness could be achieved by examining its impact across diverse player samples, impact across diverse player samples, including athletes of different age groups, competitive levels, playing positions, and tactical backgrounds.

## Conclusion

This study underscores the potential of video-based priming as an accessible and impactful tool for enhancing performance in youth football. The findings demonstrate that exposure to priming videos focusing on offensive and creative strategies can influence player behavior during SSGs. Specifically, the OFFENSIVE priming condition encouraged players to maintain stable inter-player distances, facilitating progressive ball possession and improved passing performance. In contrast, the CREATIVE priming condition led to increased spatial exploration and variability in proximity to defenders, fostering more opportunities for novel shooting attempts. However, it did not enhance creative passing or dribbling, likely due to the misalignment between the individually focused priming stimuli and the collective nature of the SSGs.

From a practical perspective, coaches can leverage video-based priming to enhance performance in different areas of the game. For offensive strategies, coaches may use videos that emphasize collective ball possession to inspire passing decision-making and execution, while creative priming videos may encourage players to develop new shooting patterns and improve spatial exploration.

## Data Availability

In order to protect the subjects’ confidentiality and privacy, data are only available upon request. Interested researchers may contact the board from the Research Center in Sports Sciences, Health Sciences, and Human Development to request access to the data (cidesd.geral@utad.pt).
